# Marker of endothelial dysfunction may improve the early detection of insulin resistance and subclinical atherosclerosis

**DOI:** 10.1186/1758-5996-7-S1-A113

**Published:** 2015-11-11

**Authors:** Bianca de Almeida Pititto, Fernando Flexa Ribeiro Filho, Marcio S Bittencourt, Isabela M Bensenor, Paulo A Lotufo, Sandra R G Ferreira

**Affiliations:** 1 UNIFESP, São Paulo, Brazil

## Background

Cardiovascular disease is still the leader cause of disability adjusted life yrs. in many developed and developing countries. Better identification of at-risk individuals is still pursued to improve preventive strategies.

## Objective

This study evaluated whether determination of E-selectin concentrations could identify gradual deterioration of cardiometabolic risk profile or subclinical atherosclerosis in individuals at low-to-moderate risk included in the Brazilian Longitudinal Study of Adult Health – ELSA-Brasil.

## Materials and methods

A sample of 998 individuals from the ELSA-Brazil (35-54 yrs.) without cardiovascular disease or diabetes was stratified according to E-selectin tertiles. Traditional risk factors, inflammatory markers and categories of coronary artery calcium (CAC) score were evaluated across the tertiles by ANOVA or chi-squared test. In linear regression models, associations of E-selectin levels with insulin resistance index, adjusted for age, sex and adiposity were tested.

## Results

The mean age of the participants was 45.8 (SD4.9) yrs. and 55% were women. Mean values of age, anthropometric data, biochemical variables and inflammatory status (assessed by IL-6/IL-10 and TNF-α/IL-10) increased across E-selectin tertiles. Also, a gradual deterioration of the cardiometabolic profile was reflected by increments in frequencies (95% CI) of BMI ≥25 kg/m2 [53.7%(48.5-58.8), 61.0%(56.1-66.5) and 64.2%(59.0-69.4), p=0.019], hypertension [18.0%(14.1-22.8), 19.8%(15.4-24.6) and 24.8%(20.4-29.9), p=0.048], pre-diabetes [62.5%(57.4-68.3), 63.1%(58.4-69.6) and 73.8%(68.8-78.3), p=0.003] and hypertriglyceridemia [22.4%(17.9-27.2), 27.3%(22.5-32.8) and 33.4%(28.3-38.5), p=0.013]. Insulinemia and HOMA-IR were independently associated with E-selectin concentration. A greater proportion of individuals with CAC score different from zero was found in the third tertile when compared with the first and second tertiles (16.1% versus 11%, p=0.04, respectively).

## Conclusions

Direct associations of E-selectin with traditional risk factors slightly above their normal ranges, components of the metabolic syndrome, insulin resistance and CAC score different from zero suggest that this biomarker may be indicating initial atherogenic process.

